# Sump Syndrome: A Rare Complication of Choledochojejunostomy

**DOI:** 10.7759/cureus.89481

**Published:** 2025-08-06

**Authors:** Nivedita, Prasanta Debnath, Rahul Samanta, Pradeepta Sethy, Habung Mobing

**Affiliations:** 1 Gastroeneterology, Medica Superspecialty Hospital, Kolkata, IND; 2 Gastroenterology, Topiwala National Medical College (TNMC) and BYL Nair Charitable Hospital, Mumbai, IND; 3 Gastroenterology, Medica Superspeciality Hospital, Kolkata, IND; 4 Gastroenterology, Medica Superspecialty Hospital, Kolkata, IND

**Keywords:** choledochodudenostomy, choledochojejunostomy, ercp, hepaticojejunostomy, sump syndrome

## Abstract

Before the period of endoscopic retrograde cholangiopancreatography (ERCP), individuals with biliary tract diseases would undergo side-to-side choledochoduodenostomy, and sump syndrome used to develop as a complication of this procedure. There is retention of bile along with debris or calculi, and refluxed duodenal contents in the common bile duct, which leads to biliary and pancreatic complications.

This syndrome's pathophysiology often results when the distal common bile duct below the anastomosis becomes a blind pouch (*sump*), leading to stasis of bile, food debris, and bacteria, which can lead to obstruction and infection.

Here, we present a case of sump syndrome as a rare complication of choledochojejunostomy. A patient with a history of post-choledochojejunostomy and redo hepaticojejunostomy, along with recurrent cholangitis, presented with abdominal pain, and initial radiological investigations suggested a pancreatic cyst. However, further endoscopic evaluation was done and revealed it to be a case of sump syndrome. ERCP followed by biliary sphincterotomy was done as a part of further management.

## Introduction

Before the era of endoscopic retrograde cholangiopancreatography (ERCP), patients with biliary tract diseases would undergo side-to-side choledochoduodenostomy, and sump syndrome would occur as one of the complications of this procedure. Choledochoduodenostomy is a surgical procedure in which an anastomosis is created between the duodenum and the common bile duct, whereas in choledochojejunostomy, an anastomosis is made between the jejunum and the common bile duct. Similarly, hepaticojejunostomy (HJ) is a surgically created communication between the hepatic duct and the jejunum using a Roux-en-Y limb.

Sump syndrome is an uncommon condition with an incidence of 0.4%-15.7% after choledochoduodenostomy [1]. Sump syndrome is very rare with choledochojejunostomy and HJ.

In a retrospective analysis of 30 cases of sump syndrome, the median interval between endoscopic sphincterotomy and the onset of symptoms was five years, while the median interval between surgery and diagnosis was six years [2].

Sump syndrome occurs many years after the original choledochoduodenostomy procedure. The clinical features usually manifest with ascending cholangitis mainly in the form of abdominal pain, fever, and jaundice. ERCP further confirms the diagnosis, and patients recover rapidly following sphincterotomy, antibiotic therapy, and endoscopic debris removal [3].

The segment of the common bile duct between the biliary-enteric anastomosis and the Ampulla of Vater functions as a sump, serving as a stagnant reservoir for debris, stones, and static bile. This stasis can lead to inflammatory or infectious changes in the biliary tree, with patients typically presenting with fever, jaundice, vomiting, abdominal pain, and tenderness [4]. Medical and surgical interventions are needed to combat the condition, as it can be fatal to the patient. Sump syndrome is a rare but serious complication of choledochoduodenostomy.

## Case presentation

Here, we present the case of a 45-year-old male patient who presented with abdominal pain for the past four to five days. The pain was dull in nature, gradual in onset, located in the epigastric region, non-radiating, and relieved only by analgesics.

The patient had been experiencing abdominal pain for the past month, for which he was admitted to a local hospital and managed conservatively. He had a history of choledochal cyst excision, cholecystectomy, and choledochojejunostomy performed in 1996, 28 years ago. Later, he developed hepatolithiasis and a large choledochal cyst stone, followed by repeated episodes of cholangitis in 2016. He subsequently underwent excision of the residual choledochal cyst, left hepatectomy, and HJ in 2017. In 2023, he developed an HJ stricture with recurrent cholangitis, for which a redo HJ was performed.

On examination, vital signs were stable; however, abdominal examination revealed mild tenderness in the epigastric region. Blood reports revealed raised CRP (Table 1).

**Table 1:** Routine tests. Hb, hemoglobin; TLC, total leukocyte count; PLT, platelet count; CRP, C-reactive protein; TB, total bilirubin; SGOT, serum glutamic-oxaloacetic transaminase; SGPT, serum glutamic-pyruvic transaminase; ALP, alkaline phosphatase; GGT, gamma-glutamyl transferase

Test	Result	Unit	Range
Hemoglobin	12.4	gm/dL	13.5-18
TLC	8,080	cells/µL	4,000-10,500
Platelet	1.5	lakhs/µL	1.5-4.5
CRP	18	mg/L	0-5
Bilirubin (total)	0.9	mg/dL	0.2-1.3
SGOT	34	U/L	17-59
SGPT	42	U/L	21-72
ALP	170	U/L	38-126
GGT	191	U/L	15-73
Albumin	4.4	g/dL	3.5-5
Urea	33	mg/dL	10-50
Creatinine	1.23	mg/dL	0.7-1.5
Sodium	141	mEq/L	137-145
Potassium	4.7	mEq/L	3.5-5.3

**Figure 1:**
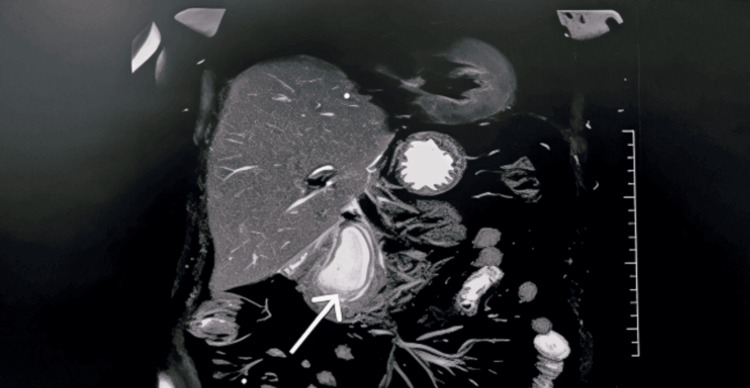
MRCP showing an elongated, well-defined cystic lesion in the region of the pancreatic head, suggestive of a pancreatic cyst. MRCP, magnetic resonance cholangiopancreatography

Serum alkaline phosphatase (ALP) and gamma-glutamyl transferase (GGT) were also raised. Magnetic resonance cholangiopancreatography (MRCP) revealed post-cholecystectomy and HJ status with prior left hepatectomy, along with an elongated, well-defined cystic lesion in the region of the pancreatic head measuring 50 × 35 × 25 mm. A provisional diagnosis of a pancreatic cyst was made. Central intrahepatic biliary radicles were mildly prominent (Figure 1).

**Figure 2:**
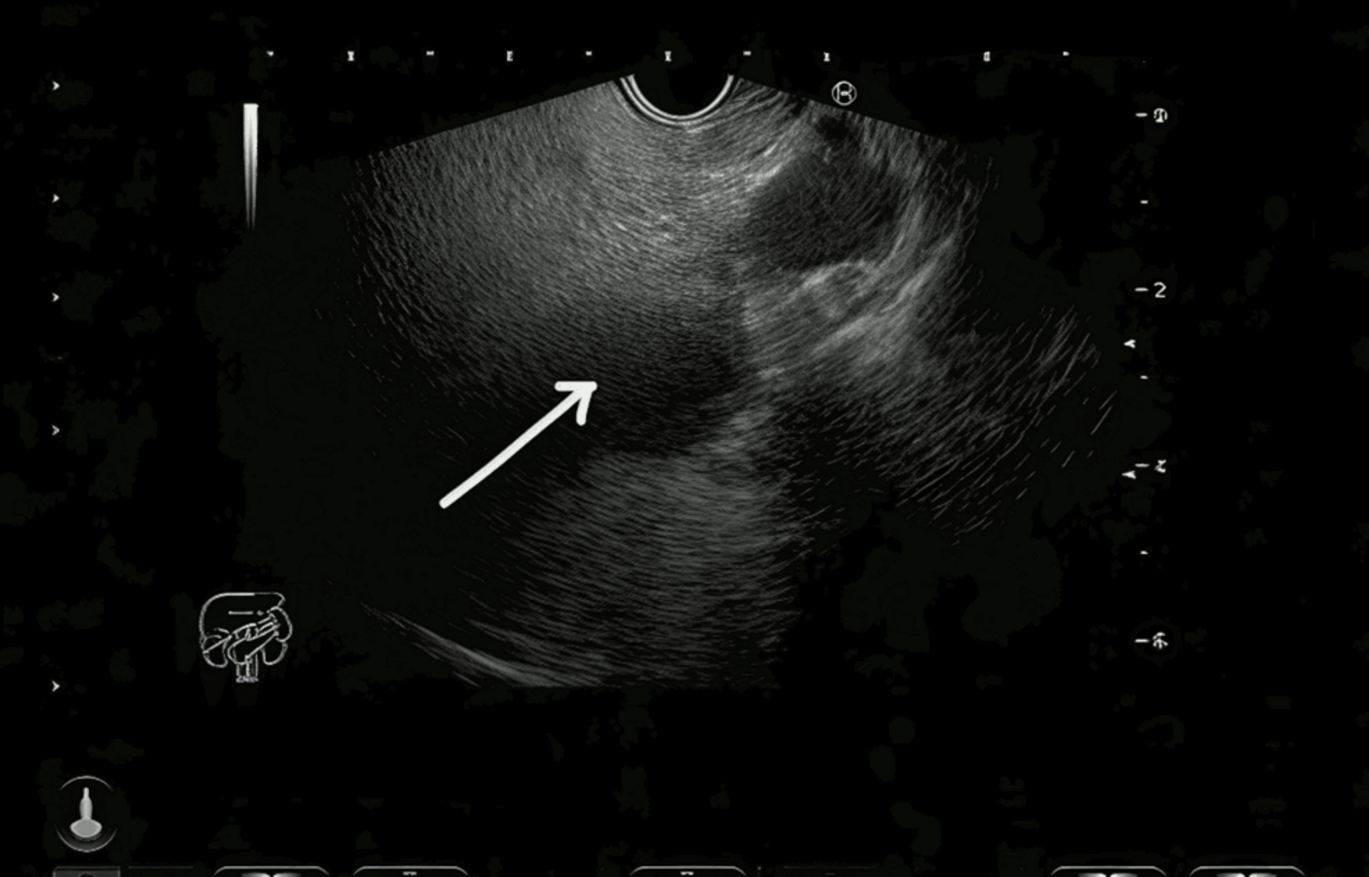
EUS showing a dilated distal common bile duct (CBD) remnant. EUS, endoscopic ultrasound

**Figure 3:**
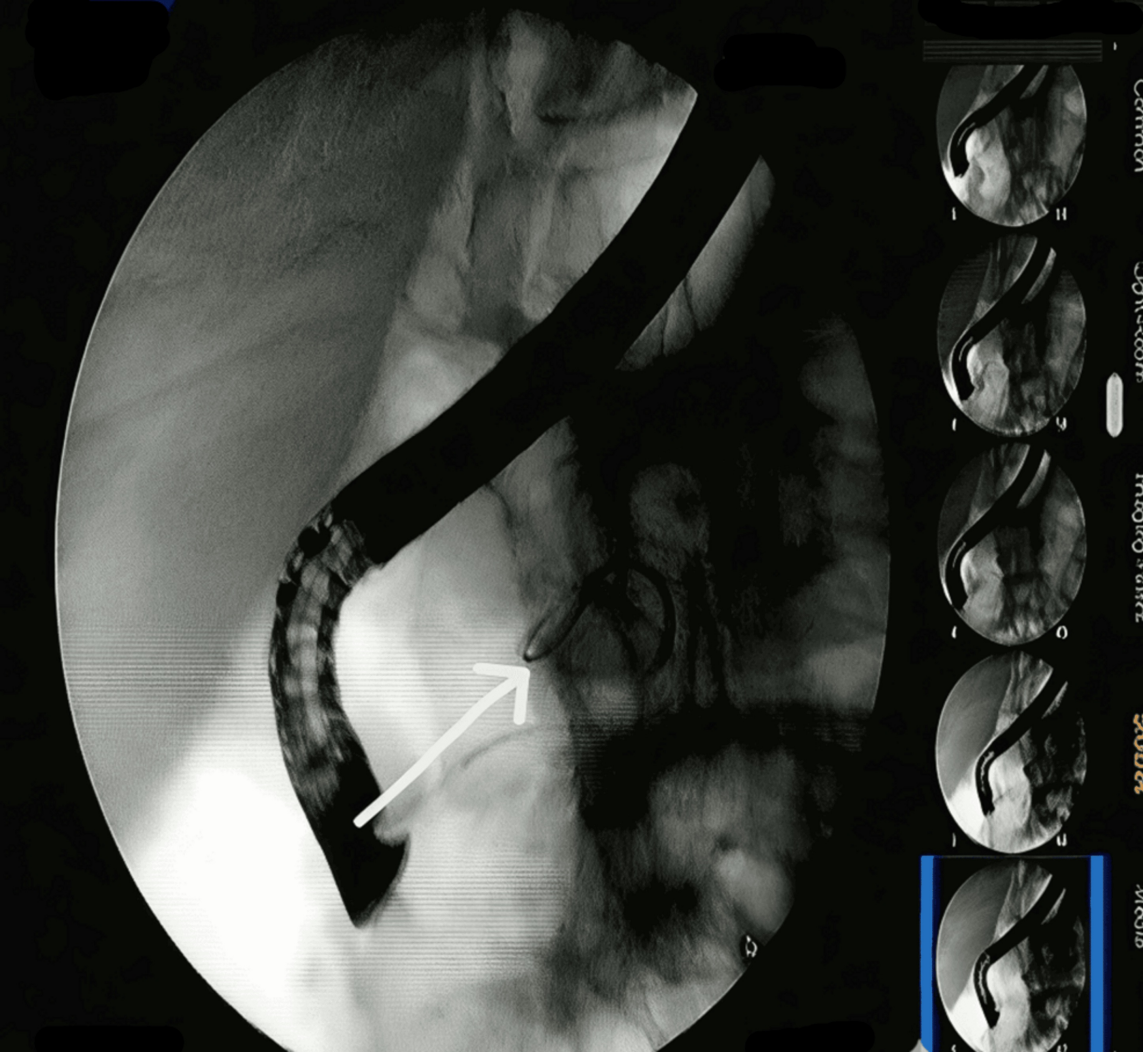
ERCP cholangiogram showing the guidewire coiled within the dilated distal common bile duct (CBD) stump. ERCP, endoscopic retrograde cholangiopancreatography

Endoscopic ultrasound (EUS) was performed as part of the further evaluation of the cystic lesion. It revealed a fatty pancreas, post-HJ status, and a dilated CBD remnant (Figure 2). Given the EUS finding, ERCP was done. The cholangiogram showed a dilated distal CBD stump (Figure 3). Biliary sphincterotomy was performed. Following ERCP and sphincterotomy, the patient showed symptomatic improvement. He also received antibiotics and was later discharged in a hemodynamically stable condition.

## Discussion

Sump syndrome is a rare, chronic complication that occurs in patients who have undergone a side-to-side choledochoduodenostomy, a surgical procedure that was commonly used to treat biliary tract disease before the advent of ERCP. Endoscopic sphincterotomy with balloon clearance of stones and debris is the treatment of choice [5].

The last procedure performed on this patient was HJ. In a study, it was seen that 20% of choledochoduodenostomies ultimately required conversion to HJ due to the development of complications [6]. Prevalence of sump syndrome in hepaticoduodenostomy is also about 10% [7].

In the study by Sather et al., a patient with a history of laparoscopic cholecystectomy and multiple ERCPs for recurrent choledocholithiasis and cholangitis underwent an end-to-side Roux-en-Y choledochojejunostomy for recurrent primary choledocholithiasis and later developed abdominal pain as a manifestation of sump syndrome [8].

In this case, a patient with a history of choledochojejunostomy and redo HJ, along with recurrent episodes of cholangitis, presented with abdominal pain and was found to have sump syndrome on evaluation. Therefore, in patients with a bilioenteric anastomosis who present with abdominal pain, the possibility of sump syndrome should always be considered and requires thorough evaluation.

## Conclusions

Despite being an uncommon complication of choledochoduodenostomy and choledochojejunostomy, sump syndrome might manifest years after the procedure. Both choledochoduodenostomy and choledochojejunostomy have been replaced by ERCP in recent years. Sump syndrome usually presents with pain or fever, and it is crucial to evaluate for radiological findings, especially after choledochojejunostomy and hepaticojejunostomy procedures, to rule out other biliopancreatic pathology.
